# Interplay of Mitochondrial Dynamics, Nutrition, and Vitamins: Evidence From Experimental and Human Studies With Implications for Women’s Health

**DOI:** 10.1155/omcl/8685788

**Published:** 2026-06-26

**Authors:** Kunjal Kiran Pai, Shambhavi Shetye, Rakshith Patil, Shravya Acharya, Kishan S. Kulal, Ajeetkumar Patil, Saritha U. Kamath

**Affiliations:** ^1^ Department of Medical Laboratory Technology, Manipal College of Health Professions, Manipal Academy of Higher Education, Manipal, 576104, Karnataka, India, manipal.edu; ^2^ Department of Orthopedics, Kasturba Medical College Mangalore, Manipal Academy of Higher Education, Manipal, Karnataka, India, manipal.edu; ^3^ Manipal Institute of Applied Physics, Manipal Academy of Higher Education, Manipal, 576104, Karnataka, India, manipal.edu

**Keywords:** mitochondria, nutrition, vitamins, women’s health

## Abstract

**Background:**

Nutrition is a key modifiable factor supporting mitochondrial health and is essential for ovarian function and women’s health across the life course. From menarche to menopause, mitochondrial efficiency underpins physiological balance. The menopausal transition is particularly critical, as hormonal and neuroendocrine changes are associated with impaired mitochondrial function and increased risk of age‐related disorders.

**Aim:**

This review aimed to systematically review and synthesize the available evidence on mitochondrial function across in vitro, animal, and human studies and to evaluate the potential protective role of vitamins and nutrients in maintaining mitochondrial health, with attention to sex‐specific findings.

**Methods:**

A systematic search was conducted across multiple electronic databases. Forty‐six eligible studies were identified and critically reviewed for evidence on mitochondrial function, sex‐based differences, and nutritional influences.

**Results:**

Mitochondrial dysfunction may contribute to the pathophysiology of age‐related disorders, including osteoporosis, cardiovascular disease, neurodegenerative conditions, and cancer. Nutritional factors are crucial for preserving mitochondrial integrity. Vitamins C, E, and D, NAD + precursors such as nicotinamide riboside, coenzyme Q10, MitoQ, fucoxanthin, and cabergoline reduce oxidative stress, enhance mitochondrial biogenesis, support electron transport chain activity and ATP production, and maintain redox balance. These actions promote mitochondrial resilience and cellular energy metabolism. Evidence further indicates that women, particularly during the menopausal transition, exhibit heightened vulnerability to mitochondrial dysfunction, highlighting the relevance of nutrition‐based interventions.

**Conclusion:**

Optimizing dietary intake of vitamins, antioxidants, and mitochondrial cofactors is a cost‐effective, accessible strategy to support mitochondrial health and reduce age‐related disease risk in women.

## 1. Introduction

Nutrition is a modifiable risk factor that plays a significant role in promoting overall well‐being and preventing disease risk throughout the lifespan, standing as a cornerstone for health and development, as emphasized by the World Health Organization (WHO) [1, 2]. Although global undernutrition remains a significant issue, there is growing recognition of the widespread challenges posed by deficiencies in vitamins and other essential nutrients [3], particularly among women experiencing unique physiological transitions such as menstruation, pregnancy, lactation, and menopause [3, 4]. Estimating the burden of micronutrient deficiencies in women across their lifespan presents unique challenges, especially given the impact of hormonal transitions, such as menstruation, pregnancy, lactation, and menopause, on nutritional needs. The micronutrient status of women not only influences their own health but also has intergenerational consequences, affecting fetal development, child growth, and long‐term health outcomes in subsequent generations [5]. Population‐representative biomarker data on micronutrient status are limited and mainly focus on women of reproductive age, with scarce information for adolescents, pregnant and lactating women, and those in peri‐ and post‐menopausal stages. Additionally, assessments often focus on only a limited number of micronutrients, despite the common coexistence of multiple deficiencies. These combined deficiencies during key hormonal transitions may influence female physiology and either exacerbate or mitigate health outcomes; however, data on their specific impacts remain limited [1, 6, 7].

Micronutrients, particularly vitamins, play an important role in maintaining overall physiological processes, including mitochondrial function. Mitochondria, in turn, influence ovarian function not only through energy metabolism but also via epigenetic modifications that regulate reproductive health [8, 9]. This highlights their significant role in female physiology. A decline in mitochondrial activity is strongly linked to diminished oocyte quality, with clinical manifestations ranging from mild to severe [10]. Since oocyte development and maturation demand an optimal energy supply, they are critically dependent on mitochondrial energy metabolism, which is strongly influenced by nutrient intake, its bioavailability, nutrient absorption, energy production, and cellular redox balance. Mitochondria–nucleus communication is central to regulating cellular adaptability, energy metabolism, health, and longevity [11, 12]. Developing oocytes are nurtured within ovarian follicles, which play a central role not only in supporting oocyte maturation but also in producing and secreting essential reproductive hormones. Apart from its central reproductive functions, the ovary is recognized as one of the earliest aging organs [13]. Despite medical advancements that have extended female life expectancy to nearly 80 years, ovarian aging typically begins around 35 years of age, with menopause occurring at ~50 years. As a result, women spend a considerable portion of their lifespan with reduced fertility [14]. Ovarian aging extends its impact far beyond reproductive capacity, influencing a wide spectrum of age‐related health conditions. Studies show that ovarian aging is associated with at least 22 systemic effects, including metabolic syndrome, cardiovascular diseases, osteoporosis, various cancers, and neurological or cognitive disorders [15]. In addition to age and genetic predisposition, several environmental factors significantly influence ovarian reserve [16]. Nutrition is a pivotal factor throughout the female lifespan, profoundly influencing reproductive milestones, including the onset of menarche, maintenance of fertility, and timing of menopause. The nutritional environment shapes physiological processes that govern reproductive function, with dietary quality and nutrient availability modulating the onset of puberty, ovarian health, and the cessation of reproductive capacity [17, 18]. Although mitochondria are increasingly recognized as central regulators of cellular energy metabolism and oxidative stress, significant gaps remain in understanding how mitochondrial dynamics specifically interact with nutritional and vitamin‐related factors to shape women’s health across the lifespan. Much of the current research on mitochondrial biology lacks a sex‐ and gender‐specific focus, often overlooking the complexity of female physiology, including hormonal fluctuations. Furthermore, tissue‐specific and age‐related variations in mitochondrial function are incompletely characterized in women, particularly in relation to nutritional status and micronutrient intake. These gaps limit our ability to elucidate the mechanisms underlying female‐specific disease susceptibilities and reproductive aging. Despite emerging evidence linking nutrition and vitamins to mitochondrial function, a comprehensive synthesis of this interplay in the context of women’s health is still lacking, underscoring the need for systematic investigation to inform targeted nutritional and therapeutic strategies.

## 2. Aim and Objective

To systematically review and synthesize the available evidence on mitochondrial function across in vitro, animal, and human studies, and to evaluate the potential protective role of vitamins and nutrients in maintaining mitochondrial health, with attention to sex‐specific findings.

## 3. Method

The methodology for the present scoping review was adapted from the framework outlined by Arksey and O’Malley framework [19]. We administered the following five steps to map Mitochondrial Dynamics in Women’s Mitochondrial Health.

1. Identifying the research question.
2. Identifying the appropriate studies.
3. Selection of studies relevant to the research question.
4. Charting the data.
5. Collating, summarizing, and reporting the results.

The results of this scoping review are reported in accordance with the Preferred Reporting Items for Systematic Reviews and Meta‐Analyses extension for Scoping Reviews (PRISMA‐ScR) guidelines.

Step 1: Identifying the research question

1. To explore the extent and type of studies conducted across populations that address mitochondrial function, with a special emphasis on understanding women’s unique susceptibility to mitochondrial dysfunction.
2. To map existing evidence on the protective role of vitamins and nutrients in maintaining mitochondrial health across various disorders.
3. To identify nutritional interventions with potential for improving mitochondrial health in women and preventing disease progression.

Step 2: Identifying the appropriate studies:

Three independent reviewers (KKP, SS, and SA) conducted the initial screening of articles retrieved from PubMed, Scopus, and Web of Science databases. Titles and abstracts will be assessed for eligibility according to the inclusion and exclusion criteria. In cases of disagreement or uncertainty during the screening process, consensus was achieved through discussion with a KSK and SKU.

Step 3: Selection of studies to answer the relevant question

**Eligibility criteria:**

We included human studies involving females of any age (from menarche through peri‐ and postmenopause) that reported outcomes related to mitochondrial function or nutrition. Eligible exposures included nutritional factors such as vitamins, antioxidants, mitochondrial cofactors, and precursors, either as dietary intake, supplementation, or baseline nutrient status. Primary outcomes were direct or indirect measures of mitochondrial function, including oxygen consumption, ATP production, electron transport chain activity, mitochondrial DNA (mtDNA) copy number, biogenesis markers, or oxidative stress biomarkers. Secondary outcomes included age‐related disorders such as osteoporosis, neurodegeneration, cardiovascular disease, and cancer. We considered randomized controlled trials, non‐randomized interventions, cohort, case–control, and cross‐sectional studies.

Step 4: Charting of Data

The primary authors have extracted and charted data based on the following variables: author, year, country of origin, study design, sample size, participants’ demographics, intervention/exposure, outcome, method of outcome measurement (laboratory test), result, conclusion, and limitations.

Step 5: Results & Discussion

**Figure 1:**
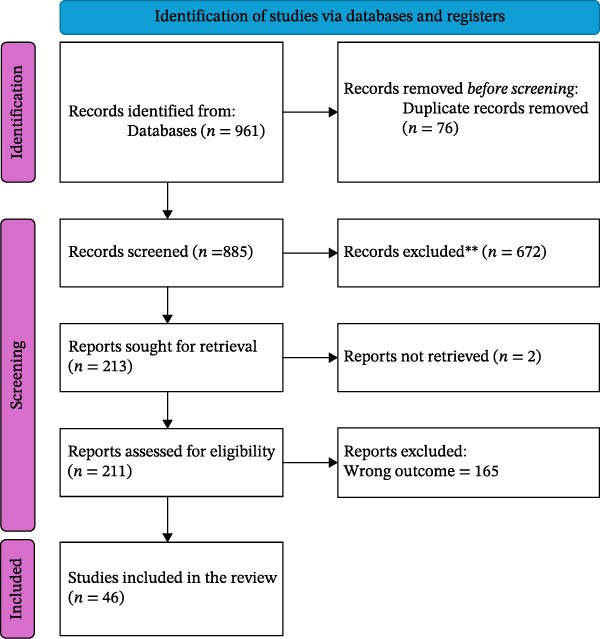
PRISMA screening chart.

The initial search across PubMed, Scopus, and Web of Science yielded 961 records. After removing duplicates, 885 records remained. These were screened based on titles and abstracts, resulting in the exclusion of 672 records deemed irrelevant. The full texts of 211 articles were assessed for eligibility; 46 studies met the inclusion criteria and were included in the final review (Figure 1).

a. Mitochondrial involvement in various disorders

Mitochondria are integral to more than just energy production; they serve important regulatory roles in cellular metabolism, redox balance, and cell survival. With advancing age, including during ovarian aging, mitochondrial function and integrity decline, leading to disruptions in energy production and an accumulation of oxidative stress. These alterations contribute to impaired cellular homeostasis and damage, fueling the development of various metabolic, reproductive, neurological, and systemic diseases that disproportionately affect women throughout their lives [20, 21]. Thus, we explored the pathophysiology and biochemical pathways through which mitochondrial health disruption could lead to age‐related metabolic, neurodegenerative, and other disorders among women. A total of 28 articles addressing various disease types were identified and included to map the evidence. The details are provided in Table 1. The study populations varied widely, with a subset of studies conducted in cell lines, including human granulosa cells [30], primary human aortic endothelial cells [33], Papillary thyroid cancer and non‐papillary thyroid cancer cells [34], breast cancer cell lines [35], brain endothelial cells [41], *Mus musculus* RAW 264.7 macrophage cells [42], thyroid follicular epithelial cells [52], and cytotrophoblasts [56]. Thirteen studies specifically focused on postmenopausal populations [22, 24, 29, 36, 40, 45, 46, 51, 53, 54], six on premenopausal women [31, 32, 34, 40, 45, 55], and two on the perimenopausal population [40, 45]. The included studies examined a wide range of conditions in which mitochondrial dysfunction was implicated. A woman’s life spans from menarche through the reproductive years to menopause, a transition that brings profound physiological changes and predisposes her to lifestyle‐related complications. Our aim was to examine the role of mitochondria throughout these stages. Emerging evidence indicates that mitochondrial alterations are one of the key contributors to a range of disorders associated with aging. The role of mitochondria has been evaluated across at least 23 distinct domains encompassing a broad range of physiological and pathological conditions. These include muscle capillarization and angiogenesis [22], obesity [24, 56], paradoxical and objective insomnia [29], acute hyperinsulinemia [31], uterine leiomyomas [32], cardiovascular function [33], papillary thyroid cancer [34], breast cancer [35, 40, 53, 54], pelvic organ prolapse [36], Alzheimer’s disease (AD) [45], cognitive frailty [55], thyroid cancer [52], vascular endothelial function [51], rheumatoid arthritis [57], urothelial carcinoma [58], endometriosis [59], muscle morphology and strength gain [46], estradiol and osteoclast differentiation [42], brain structure, connectivity, and energy metabolism [39], cerebrovascular pathophysiology [41], skeletal muscle gene expression [37], and polycystic ovarian syndrome (PCOS) [60]. Mitochondria serve many vital functions, and alterations in their performance critically influence the conditions discussed. For example, during aging, reduced oxygen delivery to mitochondria limits their ability to generate energy, impairing muscle function and regeneration. Similarly, postmenopausal women face additional challenges, as changes in adipose tissue composition, glucose metabolism, and chronic inflammation increase reactive oxygen species (ROS) production. This oxidative stress further damages mitochondria and contributes to undesirable weight gain during this stage [22, 24, 56]. During the menopausal transition, significant changes occur in brain structure, connectivity, and energy metabolism, particularly in women carrying the APOE‐4 allele, which is a known genetic risk factor for AD. Increased deposition of beta‐amyloid plaques has been observed in peri‐ and postmenopausal women with this allele, suggesting that the risk for AD begins as early as the perimenopausal phase. Estrogen, which normally helps reduce mitochondrial superoxide production and enhances the activity of antioxidant enzymes such as mitochondrial aconitase, plays an essential neuroprotective role. Despite the quantity of mitochondria remaining relatively stable across the menopausal transition, their respiratory function declines, as evidenced by decreased cytochrome c oxidase (COX) activity, which drops from ~57% in premenopausal women to 30% in perimenopausal women [39, 45]. This reduction in mitochondrial respiratory efficiency likely contributes to diminished neuronal energy metabolism, increasing vulnerability to neurodegenerative processes linked with AD. Estrogen plays a significant role in protecting cardiovascular function, extending beyond its well‐known effects on the cerebrovascular system. It protects blood vessels by activating mitochondrial antioxidant genes, thereby mitigating oxidative damage and preserving vascular health. This estrogen‐driven enhancement of mitochondrial antioxidant capacity helps maintain the integrity and proper function of blood vessels. However, during the menopausal transition, the decline in estrogen levels compromises this protective mechanism, leading to increased oxidative stress and mitochondrial dysfunction in the vascular system. This loss of estrogen‐mediated mitochondrial defense contributes significantly to vascular aging and increases the susceptibility to cardiovascular diseases in postmenopausal women [33]. Detailed pathophysiological mechanisms underlying the various conditions studied are summarized in Table 1. This table highlights the multifaceted role of mitochondrial signaling in maintaining metabolic health and how its dysfunction contributes to disease progression across these conditions.

**Table 1:** Mitochondrial involvement in various disorders.

Study ID	Study type	Country	Population	Condition studied	Mitochondrial aspect studied	Proposed pathway of mitochondrial Involvement	Key findings
Gliemann et al. [22]	Human Trial	Denmark	• Total number of participants:41 • Healthy Postmenopausal women of age 61 ± 4 years.	• Muscle Capillarization. • Angiogenesis	• Mitochondrial oxidative phosphorylation‐related proteins: Complexes I, II, III, IV, and V. • The activities of mitochondrial complexes I, II, and V were significantly higher in the very active group than in the sedentary group.	• Aging and inactivity reduce how well oxygen delivery matches the muscles’ ability to use it for energy [23]. • As people age, the function of blood vessels can decline, which may limit the amount of oxygen reaching the mitochondria. • Since mitochondria rely on oxygen to produce energy, this limitation slows down the muscles’ ability to increase oxygen use during activity. One key factor influencing oxygen delivery to mitochondria is the density of capillaries in the muscle tissue, as these tiny blood vessels determine how effectively oxygen can diffuse to the mitochondria for energy production.	• Physical activity is associated with higher skeletal muscle exercise & mitochondrial capacity in postmenopausal women. • The study found that very active postmenopausal women had higher skeletal muscle mitochondrial protein content compared with moderately active and sedentary women. They also showed greater muscle capillary density and capillary‐to‐fiber ratio, which supported faster increases in femoral arterial blood flow and oxygen uptake at the onset of exercise.
Farinha et al. [24]	Human Trial	Brazil	• Postmenopausal women aged 45–64 years. • Total number of participants: 35	Obesity	Mitochondrial parameters measured: • Electron transport chain enzymes. • Reactive oxygen species production • Methyl‐tetrazolium (MTT) reduction levels. • Reduced (GSH) and oxidized glutathione (GSSG) content. • Superoxide dismutase isoforms.	• Elevated glucose levels, altered adipose tissue composition, chronic inflammation, and increased endothelial ROS production contribute to excess body weight and oxidative stress, promoting the development of disorders such as cardiovascular disease [25]. • Regular physical activity in postmenopausal women induces biochemical adaptations, including reduced glucose and triglyceride levels, increased VO_2_ max, and overall improvement in cardiorespiratory fitness [26]. • During exercise, mitochondrial ROS are primarily generated at complexes I, II, and III of the electron transport chain (ETC), with ROS production influenced by ATP demand, VO_2_, and body temperature. Aerobic training has been shown to increase NADH‐oxidase activity in muscle, reflecting enhanced ETC activity, while shorter or low‐intensity exercise may not significantly alter mitochondrial ETC function in postmenopausal women [27, 28].	• An active lifestyle promotes antioxidant enzymes and mitochondrial adaptations in obese postmenopausal women.
Martucci et al. [29]	Human Trial	Italy	• Postmenopausal women aged 55–70 years. • Total number of participants: normal subjects (*n* = 51) & Insomnia patient (*n* = 38)	Paradoxical & objective insomnia	• Mitokines (generated as a result of mitochondrial stress response): 1. Fibroblast Growth Factor (FGF)‐21 2. Growth Differentiation Factor (GDF)‐15 3. Humanin (HN)	• Insomnia leads to the release of mitokines, and shorter sleep durations tend to increase mitochondrial ROS production. • There is also a significant difference in the circulating levels of FGF21 and HN in insomnia. • FGF21 and humanin (HN) are key mitokines involved in mitochondrial signaling. FGF21 regulates circadian rhythms and glucocorticoid release via action on hypothalamic and hindbrain neurons, and influences reproductive function during energy stress. • Humanin acts as a neuroprotective factor, potentially produced in response to stress to counteract mitochondrial dysfunction.	• Both paradoxical & objective insomnia condition showed similar levels of mitokines, indicating that insomnia affects mitochondrial stress‐response signaling. • Altered mitokines levels may contribute to some clinical symptoms of insomnia, highlighting a potential role of mitochondrial pathways in sleep disorders.
Yeh et al. [30]	In vitro	China	Human Granulosa cells	Mitochondrial biogenesis in the granulosa cells.	Mitochondrial markers: nuclear‐encoded NADH‐ubiquinone oxidoreductase alpha subunit 9 (NDUFA9) & mitochondrial‐encoded COX I.	• Mitochondrial biogenesis depends on coordinated expression of mtDNA and nuclear genes. • A key regulator, mtTFA, activates mtDNA transcription and replication.	• Ca^2+^ stimulation increased mtTFA mRNA in human granulosa cells, along with higher levels of NDUFA9 and COX I, indicating enhanced mitochondrial biogenesis.
Warren et al. [31]	Human Trial	USA	• 11 healthy premenopausal women were included. • Age: 24.7 ± 4.4 years	Acute hyperinsulinemia	Mitochondrial parameters assessed were: • H_2_O_2_ production and mitochondrial respirometry. • Genes related to energy metabolism & mitochondrial morphology (PDK4, UCP3, CPT1B, PGC1α, and SLN).	• Acute hyperinsulinemia induces increases in key insulin signaling proteins (IRS1, phosphorylated AKT) and mitochondrial biogenesis regulator PGC1α, reflecting an adaptive response. This is accompanied by increased production of reactive oxygen species (ROS) like hydrogen peroxide, indicating a transient oxidative stress response that may modulate mitochondrial function and signaling but not necessarily cause sustained mitochondrial impairment.	• Acute hyperinsulinemia increases hydrogen peroxide (H2O2, a type of ROS) production in skeletal muscle of healthy premenopausal women. • It also causes a decrease in coupling of mitochondrial respiration with ATP production, indicating an impact on mitochondrial efficiency.
Shaik et al. [32]	Human Trial	India	• Total number of participants: 14 • Premenopausal women were included.	Uterine leiomyomas	Mitochondrial Cytochrome b genes	• The strong increase in both ERα and MTCYB in tumors with the CC genotype suggests that estrogen effects on mitochondria, mediated by ERα, promote the development of leiomyoma tumors. • Estrogen receptor gene variation influences both estrogen receptor and mitochondrial gene expression, which together drives tumor growth through mitochondrial‐related pathways in premenopausal women’s uterine fibroids.	• Both ERα and MTCYB gene transcripts are significantly higher in leiomyoma tumors compared to normal uterine muscle tissue. • ERα levels are about 9 times higher, and MTCYB levels are about 5 times higher in tumors.
Liu et al. [33]	In vitro	China	Primary human aorta endothelial cells	Cardiovascular function	• Reactive oxygen species (ROS) • In vivo superoxide release • Mitochondrial superoxide dismutase (SOD) Activity measurement. • Intracellular ATP level • Mitochondrial membrane potential	• Estradiol (E2) boosts production of SOD2, an antioxidant in mitochondria that reduces harmful reactive oxygen species (ROS). • E2 works by helping estrogen receptors (ER) connect with another protein, Sp1, to turn on the SOD2 gene. • This increase in SOD2 reduces harmful reactive oxygen species (ROS), leading to less oxidative stress and improved cardiovascular health by preventing or reducing blood vessel damage.	• The estrogen helps protect blood vessels by turning on a mitochondrial antioxidant gene, reducing damaging molecules, and keeping the vessels healthy. • This finding points to new ways to treat heart risks after menopause. • The study suggests a potential gene therapy approach using viruses to deliver SOD2 to blood vessels, which can help postmenopausal women with low estrogen protect their cardiovascular health.
Rubio et al. [34]	In vitro	Florida	• Premenopausal women (*n* = 9) • Postmenopausal women (*n* = 11) • Cell line: Papillary thyroid cancer & non‐papillary thyroid cancer cells (control).	Papillary thyroid cancer	Mitochondrial retrograde signaling on the regulation of ER expression.	• The study highlights how menopause‐related changes in cell signaling and mitochondrial regulation affect estrogen receptor behavior, influencing thyroid cancer aggressiveness and treatment outcomes differently between pre‐ and postmenopausal women.	• MAPK/ERK and PI3K/AKT are important cell signals that help regulate estrogen receptor actions inside cells, both by rapid non‐genomic effects and by changing ER expression through posttranslational modifications. • In postmenopausal papillary thyroid cancer (PTC) cells, the AKT signaling pathway is more active. This AKT activity helps keep the estrogen receptor alpha (ERα) stable by stopping its breakdown, leading to higher ERα levels even though estrogen hormone levels are low. This allows ERα to be active without needing estrogen to bind, which can make tumors more aggressive as women age. • On the other hand, in premenopausal PTC cells, the ERK pathway is more active. This ERK activity reduces ERα’s ability to turn on genes.
Ward et al. [35]	In vitro	USA	• Breast cancer cell lines T47D	Breast cancer	• Mitochondrial Morphology • Mitochondrial axis length • Mitochondrial turnover	• Estrogen causes mitochondria to elongate and form more extensive networks, which is related to increased mitochondrial activity. • Estrogen causes mitochondria to elongate and form more extensive networks, which is related to increased mitochondrial activity. • Estrogen acts directly on mitochondria by increasing the activity of enzymes like manganese superoxide dismutase (SOD2), which reduces harmful reactive oxygen species (ROS) and protects cells from mitochondrial damage.	• RNA analysis showed that estrogen activates cancer‐related genes like MYC and the PI3K/AKT/mTOR pathway, which control tumor metabolism. • This study found that estrogen and progestin change how breast cancer cells make energy. Estrogen helps the cells break down glucose for energy, while progestin helps the cells store fat. When both hormones are given together, the cells use even more glucose. Estrogen also makes the cell’s mitochondria longer, but progestin stops this and encourages fat storage. These changes help breast cancer cells adapt their energy use to survive and grow better.
Alujević Jakus et al. [36]	Human Trial	Croatia	Postmenopausal women (*n* = 16)	Pelvic organ prolapse (POP)	Respiratory chain complexes measurement	• Reduced expression of mitochondrial respiratory chain complex II and a tendency toward lower citrate synthase and other complex levels, indicating impaired mitochondrial function. • Increased oxidative stress markers and mitochondrial apoptosis in pelvic support ligaments, contributing to tissue damage. • Increased oxidative stress markers and mitochondrial apoptosis in pelvic support ligaments, contributing to tissue damage.	• Overall, these findings suggest that mitochondrial dysfunction, oxidative damage, and reduced antioxidative capacity contribute to the pathogenesis of POP by promoting smooth muscle loss, increased apoptosis, and weakening of pelvic supportive structures.
Ronkainen et al. [37]	Human Trial	Finland	• Postmenopausal women aged 54–62 years	Effect of hormone replacement therapy on the skeletal muscle gene expression	• Mitochondrial DNA (mtDNA) copy number. • Transcripts related to succinate dehydrogenase complex (a component of the mitochondrial respiratory chain complex II). • Oxidative capacity per muscle cross‐section.	• Mitochondrial pathway was investigated mainly at the transcriptomic (gene expression) level, where they observed the downregulation of these transcripts with hormone replacement therapy (HRT). • However, mitochondrial DNA copy number and oxidative capacity per muscle area showed no significant differences between HRT users and non‐users.	• Long‐term hormone replacement therapy (HRT) use is linked to changes in muscle gene expression. • The observed changes in muscle composition and function in HRT users appear to result from enhanced regulation of the cytoskeleton, maintenance of muscle quality through control of the extracellular matrix, and a metabolic shift from relying on glucose to using fatty acids for energy.
Lempesis et al. [38]	Human Trial	UK	• Postmenopausal women (*n* = 23) of age 50–65 years	• Mitochondrial respiration in abdominal adipose tissue and femoral differentiated human multipotent adipose‐derived stem cells.	• Oxygen consumption rate (mitochondrial respiration). • OXPHOS protein expression • Mitochondrial DNA Copy number	• Mitochondrial respiration rates were lower in differentiated adipose‐derived stem cells from abdominal fat compared to femoral fat, although there were no significant differences in mitochondrial oxidative phosphorylation (OXPHOS) protein levels or mitochondrial DNA content between these depots. • Additionally, women with obesity exhibited lower OXPHOS protein expression in both adipose tissue and derived stem cells compared to women of normal weight.	• Postmenopausal women, upper body (abdominal) and lower body (femoral) adipose tissues have distinct oxidative characteristics, which do not appear to be influenced by the size of fat cells.
Mosconi et al. [39]	Human Trial	USA	• Total number of participants: 161 • Includes 30 pre‐menopausal, 57 peri‐menopausal, and 74 post‐menopausal participants.	Brain structure, connectivity, and energy metabolism across the transition phase in women’s lives.	• Energy metabolism assessment via ATP production. ATP to phosphocreatine ratios in parieto‐temporal regions.	• Menopausal transition involves the changes in brain areas responsible for higher‐order cognitive functions and occurs independently of age, APOE‐4 genotype, hormone therapy use, or hysterectomy status. • Comparisons with age‐matched male controls further indicate that these neuroimaging changes are linked to menopausal endocrine aging. • Importantly, brain biomarkers generally stabilized or improved after menopause, with cognitive preservation linked to recovery of gray matter volume and increased brain ATP production, suggesting compensatory mechanisms. • Concurrently, amyloid‐beta accumulation was notably higher in peri‐ and post‐menopausal women carrying the APOE‐4 allele, highlighting an increased Alzheimer’s disease risk beginning in the perimenopausal phase.	• This multi‐modality neuroimaging analysis reveals that the menopausal transition (MT) profoundly impacts brain structure, connectivity, and energy metabolism, establishing a neurological basis for both vulnerability and resilience during this period.
Vinothini et al. [40]	Human Trial	India	• Total number of participants: 60 • Pre and postmenopausal women	Breast cancer stage (I, II, and III)	Cytochrome C	• Breast tumor analysis revealed increased expression of Bcl‐2, Bcl‐xL, and Mcl‐1 with decreased expression of Bax, Apaf‐1, cytosolic cytochrome C, and caspases, indicating the mitochondrial involvement in the apoptosis pathway.	• The upregulation of anti‐apoptotic proteins alongside the downregulation of pro‐apoptotic proteins and caspases observed in this study was more evident in premenopausal breast cancer patients compared to those who are postmenopausal. • This difference may be attributable to the generally more aggressive nature of breast tumors in premenopausal women compared to those developing after menopause.
Razmara et al. [41]	In vitro	USA	Brain endothelial cells	Cerebrovascular pathophysiology	Mitochondrial ROS determination: • Enzyme activity of Aconitase, fumarase. • Mitochondrial aconitase to fumarase. • Mitochondrial superoxide production. • Manganese superoxide dismutase Activity.	• Mitochondrial carrier protein, cytochrome c involved in energy production, apoptosis & ROS production. • Estrogen results in a significant reduction in mitochondrial superoxide production in cultured human brain endothelial cells. • At physiological concentrations, estrogen markedly decreases mitochondrial superoxide levels while enhancing the activity of mitochondrial aconitase, an enzyme sensitive to mitochondrial reactive oxygen species, indicating a reduction in oxidative stress. • These protective effects of estrogen are mediated specifically through the estrogen receptor alpha (ERα) signaling pathway.	• Estrogen significantly reduces mitochondrial ROS production in brain mitochondria, acting as an antioxidant to protect brain cells from oxidative damage. • Estrogen increases activity of mitochondrial antioxidant enzymes such as manganese superoxide dismutase (MnSOD), enhancing the breakdown of harmful superoxide radicals. • Estrogen increases the activity of mitochondrial antioxidant enzymes such as manganese superoxide dismutase (MnSOD), enhancing the breakdown of harmful superoxide radicals.
Carvalho et al. [42]	In vitro	Portugal	*Mus musculus* RAW 264.7 cell line.	Estradiol and osteoclast differentiation	• Intracellular ATP measurement. • Mitochondrial membrane potential • Mitochondrial superoxide production • Complex I activity • Mitochondrial DNA (mtDNA) copy number measurements. • Mitochondrial metabolic phenotyping	• Cytokines such as Macrophage‐colony stimulating factor (M‐CSF) and the receptor activator of nuclear factor kappa B ligand (RANKL) are essential for osteoclast differentiation from myeloid precursor cells. • RANKL specifically enhances mitochondrial network formation, leading to increased oxygen consumption and elevated ATP production [43, 44]. • The interruption of mitochondrial function triggers several pathogenic signaling pathways, including: Increased permeability of the mitochondrial outer membrane (MOMP) and the mitochondrial permeability transition pore (MPTP), leading to the release of proteins like cytochrome c and mitochondrial DNA. • Release of mitochondrial DNA triggers inflammatory responses. • Impaired mitochondrial complexes and metabolite imbalances impact cell signaling and bone cell health. • The early phase of osteoclast differentiation is an energy‐intensive, mitochondria‐driven process promoted by RANKL. • Estradiol acutely disrupts this metabolic activation, halts mitochondrial biogenesis, and drives the cells into apoptosis through p53‐dependent mechanisms. • This highlights the important role of mitochondrial dynamics and apoptosis in the protective effect of estrogen against bone loss after menopause, and supports the rationale for targeting mitochondrial metabolism and signaling in osteoporosis prevention and therapy.	• RANKL, the key cytokine for osteoclastogenesis, very rapidly boosts mitochondrial Complex I activity, oxidative phosphorylation (OXPHOS), and ATP generation in osteoclast precursor cells. It activates: a. Oxidation of TCA cycle substrates, fatty acids, and amino acids b. Cardiolipin and cardiolipin synthase (mitochondrial membrane adaptation) c. Genes for mitochondrial biogenesis (PGC1β, TFAM) • Estradiol (E2) prevents the increase in OXPHOS, Complex I activity, and ATP production and decreases the oxidation of a wide range of energy substrates. • E2 induces mitochondrial‐dependent apoptosis in osteoclast progenitors by blocking RANKL‐driven mitochondrial metabolic adaptation, promoting p53‐dependent mitochondrial apoptotic signals, and halting precursor survival and differentiation.
Mosconi et al. [45]	Human Trial	USA	• Age: 40–60 years • Total number:43 • Premenopausal stage: 15 • Perimenopausal stage: 14 • Postmenopausal stage: 14	Alzheimer’s bioenergetics	• Protein concentration of enriched mitochondrial fraction • Cytochrome C Oxidase Vmax activity. • Citrate synthase activity.	• While mitochondrial quantity (CS activity) remains stable, mitochondrial respiratory function (COX activity) declines progressively from Premenopausal through Postmenopausal. • This decline is independent of age and APOE genotype and suggests that menopause is associated with impaired mitochondrial quality or efficiency in tissues assessed, potentially contributing to reduced cellular bioenergetics during and after menopausal transition.	• COX activity decreased by 30% in the perimenopausal group and by 57% in the menopausal group compared to the premenopausal group. • This ratio reflects mitochondrial respiratory capacity relative to mitochondrial content. It declines from Premenopausal (0.30) to Perimenopause (0.23) and Postmenopausal (0.20), indicating decreasing efficiency or quality of mitochondria during menopausal transition. • The study findings suggest that early therapeutic interventions during the endocrine aging process may be effective in preventing or reducing the development of bioenergetic deficits in women undergoing perimenopause and postmenopause.
Manfredi et al. [46]	Human Trial	Rhode Island	• Postmenopausal women (*n* = 5) • Age: 75.6 ± 4.28 years	Muscle morphology & strength gain	Mitochondrial size and its volume	• Aging is associated with a reduction in muscle fiber cross‐sectional area and thinning of microfilaments, particularly affecting type II fibers. These structural changes contribute to decreased muscle strength and function in older adults [47]. • Skeletal muscle serves as a critical site for oxidative stress induced by inflammatory cytokines, which contributes to muscle wasting [48]. The excessive production of reactive oxygen species (ROS) in muscle fibers leads to mitochondrial dysfunction, reduced ATP production, and increased protein degradation. • Mitochondrial bioenergetics play a crucial role in enhancing fat oxidation, improving insulin sensitivity, and supporting protein synthesis [49, 50]. These processes contribute to better metabolic health and muscle function, especially in aging and postmenopausal individuals.	• Long‐term resistance training is well‐established to confer significant benefits in older women, including the prevention or reversal of sarcopenia. • Improvements in muscle strength and increased mitochondrial content in postmenopausal muscle are indicative of enhanced mitochondrial bioenergetic capacity. • These adaptations contribute to improved muscle function and overall metabolic health in aging women.
Darvish et al. [51]	Human Trial	Colorado	• Late‐onset post‐menopausal women with an age at menopause ≥55 years, and normal‐onset PMW were aged at menopause of 45–54 years at menopause (*n* = 71). • Reference group: 21	Vascular Endothelial Function	Endothelial mitoROS	• Tonic mitochondrial reactive oxygen species (mitoROS)‐related suppression of endothelial function was reduced in late‐onset postmenopausal women. • Lower mitoROS‐associated oxidative stress was linked to better endothelial function in this group. • The circulating biochemical environment in late‐onset postmenopausal women appears to contribute to decreased mitoROS levels compared to normal‐onset postmenopausal women, thus preserving endothelial health.	• Brachial artery flow‐mediated dilation (FMD_BA_), a measure of endothelial function, was found to be 54% higher in late‐onset postmenopausal women compared to women with normal onset of menopause. However, the FMD_BA_ in late‐onset postmenopausal women was 24% lower than in premenopausal women, whereas the FMD_BA_ reduction in normal onset postmenopausal women was 51%, indicating better endothelial function in late‐onset postmenopausal women. • This suggests that late‐onset menopause is associated with relatively preserved endothelial function compared to normal‐onset menopause.
Hima and Sreeja [52]	In vitro	India	Normal thyroid follicular epithelial cell line	Thyroid cancer	• Intracellular ROS • Mitochondrial membrane potential	• Estrogen triggers mitochondrial ROS production. • The process links to modulation of uncoupling protein 2 (UCP2), a mitochondrial inner membrane protein that can reduce ROS production by uncoupling oxidative phosphorylation. • p53 and p38 signaling pathways, which respond to oxidative stress, show altered expression in estrogen‐treated thyroid cells; p53 is downregulated while p38 is upregulated, which affects cell survival pathways.	• Estrogen induces both intracellular and mitochondrial ROS production in papillary thyroid cancer (PTC) cells compared to normal thyroid cells. • Estrogen‐induced ROS contributes to the growth and progression of papillary thyroid cancer through regulation of UCP2, mitochondrial ROS balance, and related signaling pathways. • Highlighting the reasons for the increased frequency of thyroid cancer in females and its decline after menopause, underscoring the role of mitochondrial ROS and UCP2 as critical mechanistic factors and potential therapeutic targets
Sastre‐Serra et al. [53]	Human Trial	Spain	Postmenopausal women (*n* = 13)	Breast cancer	Mitochondrial Respiratory Chain Complexes	• Tumors with a low ERα/ERβ ratio display increased oxidative damage and elevated levels of antioxidant enzymes, uncoupling proteins (UCP), and sirtuin 3 (SIRT3), along with heightened activation of studied signaling pathways.	• Positive correlations were observed between ERα/ERβ ratio and glutathione peroxidase, mitochondrial respiratory complexes (V, III, II, IV), AKT, SAPK, and ERα. • Negative correlations were found between the ERα/ERβ ratio and carbonyl groups (markers of oxidative damage), catalase, CuZn‐superoxide dismutase, UCP5, SIRT3, and ERβ.
Adams‐Reyes et al. [54]	Human Trial	Mexico	Postmenopausal women (*n* = 6)	Breast cancer	MitoChip v2.0 sequencing	• Somatic mutations were identified in mitochondrial protein‐coding genes involved in aerobic respiration: NADH dehydrogenase (ND1, ND2, ND3, ND4, ND5), cytochrome c oxidase subunit I (CO1), and ATP synthase subunit 6 (ATP6). These mutations may alter mitochondrial oxidative phosphorylation (OXPHOS) function. • Somatic mutations were identified in mitochondrial protein‐coding genes involved in aerobic respiration: NADH dehydrogenase (ND1, ND2, ND3, ND4, ND5), cytochrome c oxidase subunit I (CO1), and ATP synthase subunit 6 (ATP6). These mutations may alter mitochondrial oxidative phosphorylation (OXPHOS) function. • Somatic mutations were identified in mitochondrial protein‐coding genes involved in aerobic respiration: NADH dehydrogenase (ND1, ND2, ND3, ND4, ND5), cytochrome c oxidase subunit I (CO1), and ATP synthase subunit 6 (ATP6). These mutations may alter mitochondrial oxidative phosphorylation (OXPHOS) function.	• The study identified 64 genetic variations. • Women with normal BMI exhibited a higher proportion of somatic mutations in protein‐coding mitochondrial genes involved in aerobic respiration compared to those with obesity. • Fewer mutations were found in the mtDNA D‐loop non‐coding region. • Obese patients showed increased polymorphisms in mitochondrial genes, some of which are known to be associated with various cancers, suggesting mitochondrial genetic variability may influence cancer susceptibility and progression within this ethnic group.
Qin et al. [55]	Human Trial	China	• Older adults (≥50 years) • Cognitive Frailty (CF): 90 (*F* = 30%) • Non‐CF: 46 (*F* = 9%)	Cognitive Frailty (CF)	• Oxygen consumption rate (OCR) in PBMCs. • Baseline OCR • ATP‐linked respiration • Proton leak • Maximal respiration • Spare respiratory capacity	• CF showed lower expression of mtDNA compared with the non‐CF group. • Baseline OCR, ATP‐linked respiration, Maximal respiration, and Spare respiratory capacity are reduced in CF. • Proton leak did not differ significantly between the 2 groups. • Carnitine Palmitoyl transferase 2 (CPT2) elevated in CF group.	• Study show that mitochondrial dysfunction is closely linked to cognitive frailty (CF), a condition marked by both physical weakness and cognitive impairment in older adults. Mitochondria, which provide the energy required for proper neuron function and brain health, show impaired activity in CF.
Phillips et al. [56]	Human Trial	US	• Women in gestational weeks 39–40. • Sample: venous blood, cord blood, cytotrophoblast.	Obesity	Mitochondrial Respiration Assay	• Mitochondrial dysfunction was observed in cytotrophoblasts isolated from the placentas of women with obesity or high BMI. • Treating cytotrophoblasts from placentae with vitamin D resulted in better mitochondrial performance, as shown by increased spare (reserve) respiratory capacity and enhanced proton leak function. This indicates improved adaptability and efficiency of mitochondria in placental cells exposed to vitamin D, which could help counteract dysfunction seen with maternal obesity.	• Normal weight women: No correlation observed between maternal plasma vitamin D level and maternal BMI. • Obese women: An Inverse correlation existed between maternal circulating vitamin D and maternal BMI. • A significant correlation exists between cord blood level of vitamin D and placental efficiency. • Maternal obesity does not affect the expression of CYP27B1 in the placenta.
Kaushal et al. [57]	Human Trial	India	• Participants with RA (n): 40 • Age: 38.95 ± 7.910 years • Female: 37 • Healthy control (n): 40 • Age: 31.73 ± 5.089 years • Female: 32	Rheumatoid arthritis (RA)	• Intracellular ROS • Enzymatic oxidative stress markers: catalase, superoxide dismutase, glutathione peroxidase. • Non‐enzymatic oxidative stress markers: reduced glutathione, lipid peroxidation. • Mitochondrial ROS • Mitochondrial Membrane Potential (MMP)	• Mitochondrial dysfunction and increased oxidative stress in polymorphonuclear neutrophils (PMNs) were assessed by measuring mitochondrial reactive oxygen species (ROS) using MitoSOX staining. • The percentage of MitoSOX‐positive cells was significantly higher in rheumatoid arthritis (RA) patients (mean 45.70 ± 24.52) compared to healthy controls (mean 24.61 ± 9.31). This indicates elevated mitochondrial superoxide production and oxidative damage in RA PMNs. • Intracellular reactive oxygen species levels, measured by median fluorescence intensity, were elevated in RA patients compared to healthy controls. • The antioxidant defenses responsible for controlling ROS, such as catalase, superoxide dismutase (SOD), and glutathione peroxidase, were diminished in RA patients. • Additionally, the levels of reduced glutathione, an important molecule for neutralizing oxidative stress, were significantly lower in individuals with RA.	• Neutrophils have important functions in rheumatoid arthritis (RA) progression, including generating oxidative stress, controlling antioxidant defenses, maintaining inflammation, and causing tissue damage. • In RA patients, neutrophils show higher oxidative stress inside the cell and mitochondria, with lower levels of antioxidant enzymes (catalase, SOD, glutathione peroxidase) and reduced glutathione. • There is also a decrease in Nrf2, a key regulator of antioxidant responses, which worsens redox balance. Elevated inflammatory cytokines (IL‐1β, IL‐6, TNF‐α) in serum further enhance immune activation, while levels of proteins MMP2 and MMP9 involved in tissue remodeling are reduced. • This complex network of factors contributes to RA development and could provide targets for diagnosis and treatment.
Palacka et al. [58]	Human Trial	Slovakia	• Participant group with UC (n): 15 (10 men + 5 women). • Age: 58–83 years • Control group (n): 15 healthy volunteers (6 men + 9 women) • Age: 35–67 years	Urothelial carcinoma (UC)	• Mitochondrial respiration in platelets. • Citrate synthase activity • Antioxidant and oxidant stress determination (co‐enzyme Q10 total, α‐tocopherol, γ‐tocopherol, β‐carotene. • Oxidative stress measurement by using TBARS (Thio barbituric acid reactive substances)	• Oxygen consumption and leak respiration linked to complex I (CI) were similar between UC patients and controls. • CI‐linked oxidative phosphorylation (OXPHOS) capacity was reduced to 74.5% in the UC group. • CI‐linked electron transfer (ET) capacity decreased significantly to −69.3% vs controls. • Combined CI and complex II (CII)‐linked ET capacity was reduced to 82.8%. • Glycerophosphate pathway respiration was significantly lower (81.4%) in UC patients (*p* = 0.020). • There was a significant reduction in mitochondrial respiration pathways linked to complex I (CI), malate, and glycerophosphate dehydrogenase complex in the platelets of the UC group.	• The study highlights altered mitochondrial function in platelets from the urothelial carcinoma participant group, showing reduced complex I‐linked oxidative phosphorylation and electron transfer capacities. • Elevated oxidative stress, indicated by increased TBARS levels, suggests mitochondrial dysfunction contributing to metabolic reprograming in UC, potentially shifting platelet metabolism from OXPHOS towards glycolysis.
Ekici et al. [59]	Human Trial	Turkey	• Group of participants with endometriosis (*n*): 10 • Age‐matched control (*n*): 10 • Age: 25–30 years • Sample type: Neutrophils and serum	Endometriosis	• Caspases 3 and 9 • Intracellular ROS • Mitochondrial Membrane Potential • Lipid peroxidation measurement • Reduced GSH & Glutathione peroxidase • TRPM2 and PARP 1 expressions • Vitamin A and Vitamin E levels	• Calcium overload caused by excessive entry through the TRPM2 channel results in the loss of mitochondrial membrane potential (depolarization) and increases the production of reactive oxygen species (ROS) inside the cell. • Lipid peroxidation levels were significantly higher in participants with endometriosis, indicating increased oxidative stress. • The antioxidant molecules glutathione (GSH) and glutathione peroxidase (GPx) were decreased in the endometriosis group, suggesting reduced antioxidant defense.	• Intracellular free calcium from calcium entry through TRPM2 activation in neutrophils was higher in endometriosis. • In endometriosis, the TRPM2 channel plays a key role in permitting calcium entry into neutrophils. • Serum Vitamin A and E concentrations were significantly lower, which increased after treatment with CBG in participants with endometriosis.
Gong et al. [60]	Human Trial	China	• Age: 22–36 years • Participant group: PCOS & Non‐PCOS group. • cell line: Granulosa cell	Polycystic ovarian syndrome (PCOS)	• Intracellular ROS • Apoptosis Assay • MMP	• The intensity of ROS in the PCOS group treated with growth hormone (PCOS‐GH) was lower than that of the PCOS control group, but no difference with the non‐PCOS group. • MMP in the PCOS‐GH group was higher than in the PCOS‐C group.	• Growth hormone reduced reactive oxygen species (ROS) production by over 50%, while simultaneously enhancing mitochondrial membrane potential (MMP) and lowering both early and late apoptosis under polycystic ovary syndrome (PCOS) conditions. • It also activated PI3K/Akt signaling.

b. Protective role of vitamins and nutrients in maintaining mitochondrial health across various disorders

Vitamins, beyond their essential dietary roles, are also important for maintaining mitochondrial health and for protecting against various disorders. Given the widespread involvement of mitochondrial dysfunction in many disease states and its impairment during ovarian aging, we aimed to map the evidence on the impact of vitamins on mitochondrial function, with a particular focus on women. A total of nine studies have been included [61–63]. Four notable studies have specifically highlighted the vital role of vitamin D in maintaining mitochondrial health across both health and disease contexts [61–64]. Two studies have examined the effects of vitamin D on overall mitochondrial function. In one study, the active form of vitamin D was shown to enhance mitochondrial fatty acid oxidation capacity in muscle tissue. In another clinical trial, supplementation with cholecalciferol significantly increased mitochondrial oxidative phosphorylation, leading to improvements in clinical symptoms such as muscle weakness and fatigue [61, 62]. These findings suggest that vitamin D plays a key modulatory role in supporting mitochondrial bioenergetics and muscle function, at both the cellular and clinical levels. In contrast to the beneficial effects observed in other studies, an in vitro study using INS‐1 β‐cells revealed that vitamin D treatment did not significantly improve mitochondrial function. However, vitamin D upregulated genes associated with beta‐cell function and markedly increased insulin secretion in response to glucose at both baseline and elevated concentrations [64]. Vitamin D is also known to inhibit cancer cell proliferation, suppress cancer stem cell growth, and beneficially modulate the tumor microenvironment [63], thereby highlighting its extended role beyond bone and mineral metabolism. Beyond vitamins, literature searches have identified several bioactive compounds and nutrients that influence mitochondrial function and overall health. For instance, a study on human fibroblast cells from patients with Friedreich’s Ataxia (FRDA) has shown that treatments with dimethyl fumarate (DMF), N‐acetylcysteine (NAC), and L‐ascorbic acid (LAA) increase frataxin (FXN) and nuclear factor erythroid 2‐related factor 2 (NRF2) expression. These proteins enhance the expression of antioxidant enzymes and reduce mitochondrial and cytosolic reactive oxygen species (ROS) levels. Additionally, combined treatment with LAA and NAC increased mitochondrial mass in these cells, while DMF treatment elevated the mtDNA copy number, indicating improved mitochondrial biogenesis and function [65]. In the context of diabetic ketoacidosis (DKA), supplementation with thiamin alone has been shown to improve oxygen consumption rate (OCR) by 16%–21%. In contrast, coenzyme Q10 (CoQ10) supplementation demonstrated a greater effect, increasing OCR by ~46%. These findings suggest that both thiamin and CoQ10 play protective functional roles in maintaining mitochondrial health, with CoQ10 exhibiting a more pronounced enhancement of mitochondrial respiratory function [66]. A detailed description of each is provided in Table 2.

**Table 2:** Protective role of vitamins in maintaining mitochondrial health.

Author/year	Country	Study design	Population	Key findings
Vernerova et al. 2025 [62]	Czech Republic	Prospective Interventional Study	Sample size: • Idiopathic Inflammatory Myopathy (*n* = 46). • Female/male: 56/11 (84%) • Age: 50.9 ± 14.7 years • Healthy Control (*n* = 67) • Female/Male: 39/7 (85%) • Age: 56.7 ± 12.4 years	• Measurement of 25(OH)D, 1,25(OH)D, VDR and CYP27B in muscle tissue and primary muscle cell. • Decreased level of the active form of Vitamin D, indicating its impairment in the Idiopathic Inflammatory Myopathy. • 25(OH)D‐ No difference was observed for this form of Vitamin D. • In differentiated muscle cells, increased VDR expression is associated with reduced mitochondrial fatty acid oxidation, as reflected by lower complete oxidation of saturated palmitic acid. • Higher VDR levels correlate with decreased clearance of palmitate via both oxidative and non‐oxidative pathways. • Additionally, elevated VDR expression is linked to a reduced relative abundance of ATP synthase (Complex V), suggesting a diminished cellular capacity for ATP production. • Increased VDR expression is positively associated with higher levels of mitochondrial complexes I and IV. • Active vitamin D positively influences mitochondrial fatty acid oxidation capacity in muscle via upregulation of CPT1, while the inactive vitamin D form shows an inverse relationship.
Nemec et al. 2021[67]	Slovakia	In vitro study	• Sample type: Differentiated skeletal muscle cells (myotubes).Groups: • Participants with IIM (*n* = 7) • Intervention type: Before training & after training. • Sedentary healthy control (*n* = 7). Gender: • Participants with IIM (*n* = 7): Male (*n* = 2) and female (*n* = 5). • Sedentary healthy control (*n* = 7): Male (*n* = 2) and female (*n* = 5).Mean age: • Before training: 52.7 ± 9.6 • After training: 53.1 ± 9.7 • Control group: 49.4 ± 9.9	• The absolute content of oxidative phosphorylation (OXPHOS) proteins in skeletal muscle remains unchanged regardless of disease state or exercise intervention. • However, consistent exercise training leads to an increase in the relative abundance of mitochondrial complexes I, II, III, and IV. • Conversely, the relative amount of ATP synthase (Complex V) decreases. • Palmitate treatment increased carnitine palmitoyltransferase‐1 (CPT1) mRNA expression in both idiopathic inflammatory myopathy (IIM) patients and healthy control muscle cells, indicating a possible upregulation of the rate‐limiting enzyme involved in mitochondrial fatty acid oxidation in response to palmitate exposure. • No significant difference in baseline PPARGC1A mRNA expression between IIM patients and healthy controls (*p* > 0.05). • Palmitate exposure increased PPARGC1A mRNA levels in myotubes derived from IIM patients, but this effect was observed only after a training intervention. • Palmitate exposure increased PPARGC1A mRNA levels in myotubes derived from IIM patients, but this effect was observed only after a training intervention. • Similarly, myogenin mRNA levels remained unchanged following chronic palmitate treatment. • Palmitate exposure induces shifts in the relative abundance of key mitochondrial OXPHOS protein complexes, particularly increasing complexes I, III, and IV while decreasing complex V (ATP synthase) in both IIM patient‐derived and healthy control myotubes. These changes suggest mitochondrial adaptations or remodeling in response to lipid stress.
Sunitha et al. 2016^68^]	India	Observational comparative study	• Sample type: Muscle tissue • Number of samples: • Control group: 1. *n* = 3, (Female = 2), Age = 15–40 years 2. *n* = 20 (Female = 5), Age = 20–35 years • Participants with dysferlinopathy: *n* = 43 (Female = 22), Age = 17–40 years • Distal Myopathy with rimmed vacuoles: *n* = 31 (Female = 10), Age = 23–40 years • Polymyositis: *n* = 24 (Female = 17), Age = 7–67 years.	• Muscle pathology analysis identified typical ragged fibers on modified Gomori trichrome (MGT) staining, with ragged blue fibers observed on SDH enzyme staining and notable loss of cytochrome c oxidase (COX) in samples from polymyositis and dysferlinopathy cases, indicating mitochondrial dysfunction. • A significant increase in the ADP/ATP ratio was observed in muscle tissue from participants with dysferlinopathy and polymyositis, indicating impaired mitochondrial energy production and bioenergetics in these muscle diseases. • Ultrastructural examination revealed mitochondrial abnormalities characterized by subsarcolemmal clustering, concentric and distorted cristae, as well as diverse mitochondrial morphologies, including vacuoles, elongated, and rounded forms in dysferlinopathy and aggregation and appearance of fragmented mitochondria in Distal Myopathy with rimmed vacuoles. • Mitochondrial proteomic analysis (*n* = 446) identified 68 proteins upregulated and 177 proteins downregulated across three muscle diseases. Of the upregulated proteins, 36 were associated with polymyositis, 29 with dysferlinopathy, and 45 with distal myopathy with rimmed vacuoles. Among the downregulated proteins, 95 were related to polymyositis, 139 to dysferlinopathy, and 139 to distal myopathy with rimmed vacuoles. Ten upregulated and 80 downregulated proteins were common to all three disease pathologies. • A significant reduction in the activity of respiratory complexes I, II, and III, along with decreased activities of Krebs cycle enzymes citrate synthase and malate dehydrogenase, as well as other enzymes, including aspartate aminotransferase and superoxide dismutase, in all three pathological conditions compared to controls.
Taneera et al. 2025 [64]	UAE	In vitro experimental study	• Cell line studied: Clonal rat β‐cells INS‐1.	• Vitamin D treatment of INS‐1 cells did not result in a significant change in cell viability compared to control cells. • Vitamin D treatment at concentrations of 20 and 40 nM significantly increased insulin secretion from INS‐1 cells at both baseline glucose (2.8 mM) and high glucose stimulation (16.7 mM) compared to controls. However, insulin content within the vitamin D treated cells remained unchanged. • Vitamin D treatment at 20 and 40 nM concentrations caused a substantial increase in vitamin D receptor (VDR) expression at both the mRNA and protein levels in INS‐1 cells. Additionally, at 40 nM vitamin D, there was significant upregulation of beta‐cell function‐related genes, including glucokinase (Gck) and insulin receptor beta (Insrb) at the mRNA level, indicating enhanced beta‐cell functional gene expression with vitamin D treatment. • Vitamin D (VD) treatment did not significantly affect mitochondrial metabolism in INS‐1 cells, as shown by MitoTracker staining and flow cytometry, which revealed no difference in fluorescence intensity between VD‐treated and control cells. In contrast, the positive control FCCP increased mitochondrial fluorescence intensity. However, VD treatment at 20 and 40 nM significantly increased intracellular calcium levels in INS‐1 cells compared to vehicle controls (*p* < 0.05). Despite these changes, VD treatment did not impact cell proliferation, with no observed differences between VD‐treated and control cells at 24 and 48 h post‐treatment. • RNA‐seq analysis using the Islet Gene View tool revealed that human pancreatic islets have the highest vitamin D receptor (VDR) expression among metabolic tissues, with α‐cells expressing higher VDR than other endocrine cells, including β‐cells. • VDR expression is significantly reduced in individuals with hyperglycemia/diabetes, suggesting a link between VD deficiency and β‐cell dysfunction that may worsen the pathophysiology of type 2 diabetes (T2D).
Elmorsy et al. 2025 [69]	Saudi Arabia	Experimental in vitro study	• Cell line studied: KGN Cell line derived from human ovarian granulosa cells.	Cytotoxicity: • Higher concentrations of mercury decreased granulosa cell viability and increased lactate dehydrogenase (LDH) leakage, indicating cytotoxicity. The effect of fucoxanthin on mercury‐induced cytotoxicity was assessed using the bromodeoxyuridine proliferation assay, where fucoxanthin at 10 µM was observed to reduce mercury‐induced cytotoxicity. Intracellular ATP levels: • Mercury exposure at concentrations of 1 and 10 µM significantly decreased intracellular ATP levels in human ovarian granulosa cells to 70.1% ± 2.7% and 49.2% ± 3.7% of control levels, respectively. In contrast, treatment with fucoxanthin at 10 µM increased ATP content to 135.6% ± 2.8% in control cells and mitigated the detrimental effects of mercury on ATP levels during co‐treatment. Mitochondrial Membrane Potential: • Fucoxanthin at 10 µM concentration increased mitochondrial membrane potential to 120.7% ± 3.5% in control granulosa cells. Mercury exposure at 1 µM and 10 µM reduced the membrane potential to 70.3% ± 3.5% and 54.2% ± 2.9%, respectively. • Co‐treatment with 10 µM fucoxanthin partially restored the membrane potential to 95.7% ± 3.7% and 73.2% ± 2.7% at these mercury concentrations, indicating its protective effect against mercury‐induced mitochondrial dysfunction. • Oxygen consumption rate (OCRs): • Mercury exposure at 1 and 10 µM significantly reduced the oxygen consumption rates (OCRs) of granulosa cells to 78.9% ± 4.3% and 55.7% ± 3.9% of control values, respectively. However, fucoxanthin co‐treatment (10 µM) increased OCRs in mercury‐exposed cells (1 & 10 µM) to 107.6% ± 3.3% and 78.2% ± 4%, demonstrating its protective effect against mercury‐induced mitochondrial respiratory impairment. • Lactate production: • Exposure of granulosa cells to mercury at concentrations of 1 and 10 µM markedly increased lactate production through anaerobic glucose metabolism to 142.5% ± 4.1 and 158% ± 3.9% of control, respectively. In cells treated with both fucoxanthin and mercury, lactate levels were lower than those observed with mercury alone, indicating a protective effect of fucoxanthin against mercury‐induced metabolic disruption. • Mitochondrial complex I: • Mercury at 1 μM decreased Complex I activity to 62.3 % ± 3.2% of control. • At 10 μM, mercury further lowered Complex I activity to 46.3% ± 3.5% of control. • Fucoxanthin at 10 μM increased Complex I activity to 122.4% ± 2.9% of control. • With both fucoxanthin and mercury, the reduction by mercury was less severe. Mitochondrial complex II: • Mercury at 1 μM reduced Complex II activity to 76.5% ± 3.4% of control. • At 10 μM, activity dropped to 66.5% ± 3.2% of control. • Fucoxanthin at 10 μM raised Complex II activity to 117.8% ± 2.8% of control. • Fucoxanthin co‐treatment helped prevent mercury’s suppression. • Mitochondrial complex III: • Mercury at 10 μM decreased Complex III activity to 68.2% ± 3.3% of control. • Fucoxanthin at 10 μM increased Complex III activity to 114.5% ± 3.1% of control. Mitochondrial Complex IV: • Mercury at 10 μM reduced Complex IV activity to 65.7% ± 3.4% of control. • Fucoxanthin at 10 μM increased Complex IV activity to 112.4% ± 2.6% of control. • Mitochondrial gene expression: • Exposure of the cell line to mercury (1 and 10 μM) significantly reduced the expression of mitochondrial electron transport chain (ETC) genes, including ND1, ND5, cytochrome b (Cy.b), cytochrome c oxidase (COI), and ATP synthase subunits 6 and 8 (ATP6/8). Treatment with fucoxanthin at 10 μM increased the expression levels of these genes, counteracting the suppressive effects of mercury on mitochondrial gene expression. Mitochondrial Membrane Fluidity (MMF): • When granulosa cells (GCs) were treated with mercury (Hg) at 1 and 10 μM for 72 h, the mitochondrial membrane fluidity (MMF) significantly increased to about 120% and 137% of normal levels. • Treatment with fucoxanthin (FX) at 10 μM lowered the MMF to about 75%. When cells were treated with both mercury and fucoxanthin, fucoxanthin helped reduce the increase in membrane fluidity caused by mercury in a dose‐dependent way. Mitochondrial membrane fatty acid composition: • Mercury increased the levels of unsaturated fatty acids like palmitoleic, oleic, linoleic, arachidonic, and docosahexaenoic acids while decreasing saturated fatty acids such as palmitic and stearic acids. When cells were treated with both fucoxanthin and mercury, fucoxanthin helped reduce these mercury‐induced changes in the fatty acid composition of the mitochondrial membrane. Mitochondrial Swelling: • Mercury exposure induced an increased rate of mitochondrial swelling, which was effectively counteracted by co‐treatment with fucoxanthin at a concentration of 10 μM. Mitochondrial membrane permeability:Exposure to mercury elevated the membrane permeability to H^+^ and K^+^ ions, whereas co‐treatment with fucoxanthin (10 μM) reduced this effect.
Edzeamey et al. 2025 [65]	UK	In vitro study	Healthy human fibroblast cell line	• Friedrich’s ataxia shows low‐level FXN gene expression, which drives disease progression. NRF2 is a transcription factor essential for activating antioxidant enzymes, whose expression is reduced under this condition. • Treatment with dimethyl fumarate, N‐acetyl cysteine, and L‐Ascorbic acid increases the mRNA expression level of FXN & NRF2 in human FRDA fibroblast. • The combination of L‐ascorbic acid (LAA) and N‐acetyl cysteine (NAC) provided the strongest protective effect, leading to a significant decrease in mitochondrial ROS (mROS) levels. All treatments were effective in reducing cytosolic ROS (cROS). • Co‐treatment with LAA and NAC increased mitochondrial mass in FRDA fibroblasts. • Dimethyl fumarate (DMF) alone enhanced mitochondrial DNA copy number. • Combinations of LAA + NAC, LAA + DMF, and NAC + DMF significantly increased mitochondrial DNA copy number in both FRDA and control fibroblasts. • Both individual treatments and their combinations elevated frataxin expression and upregulated NRF2. • In FRDA cells, higher levels of aconitase and citrate synthase activity were associated with enhanced NRF2 nuclear translocation.
Vine et al. 2024 [66]	USA	Observational study	Individuals with Diabetic ketoacidosis • *n*: 62 • Age: 45 years • Male: 53.2% Control Group • *n*: 48 • Age: 24 • Male: 20.8%	• Basal respiration (basic cell energy use) is lower in DKA patients compared to healthy people. • Maximal respiration (the highest energy cell can produce) is also lower in DKA but increases with treatment. • ATP‐linked respiration, the energy linked to making ATP (cellular energy currency), is similar in DKA and controls at baseline but improves after treatment. • Giving thiamin increased OCR by about 16%–21% in both DKA and healthy groups, meaning it helps improve oxygen use and energy production. • CoQ10 improved OCR even more, especially in DKA patients (up to 46% increase), which means it supports mitochondrial function, particularly in disease.
Li et al. 2024 [63]	China	Experimental study	Cell line: A2780 & ES‐2 Cell line of ovarian cancer	• Gene Set Enrichment Analysis (GSEA) showed that, in one aggressive ovarian cancer subtype (type II OC), two key metabolic pathways stand out: • The ferroptosis pathway (involving FTH1) is a form of iron‐dependent cell death. • The steroid biosynthesis pathway (involving CYP24A1), related to hormone metabolism. • Certain mitochondrial genes like FTH1 and CYP24A1 are upregulated in a specific aggressive OC subtype, while others (COX8C, MCCD1, UCP1, HMGCS2, GPAT2) are downregulated. • CYP24A1 is linked to increased tumor malignancy risk and worse prognosis in OC. • CYP24A1 plays a crucial enzymatic role by converting active vitamin D (1,25‐dihydroxy vitamin D) into less active metabolites (24,25‐dihydroxy vitamin D and 1,24,25‐trihydroxy vitamin D), maintaining intracellular vitamin D balance. • Vitamin D reduces cancer cell growth, cancer stem cells, and modulates the tumor microenvironment beneficially. • The Vitamin D receptor (VDR) is a nuclear receptor essential for vitamin D signaling and metabolism. • When vitamin D binds to VDR, the receptor forms a complex that enters the nucleus and attaches to vitamin D response elements (VDRE) on DNA, controlling gene expression to influence cell functions. • CYP24A1 indirectly reduces VDR signaling by promoting degradation of the active form of vitamin D (1,25(OH)2D), which affects vitamin D‐dependent gene regulation and cellular effects [70, 71].
Sinha et al. 2013 [61]	UK	Clinical trial	• Total participants: 12 with vitamin D level < 15 nmol/L. • Age: 18.1–50.4 years • Female participants (*n*): 5	• Supplementation of cholecalciferol has increased mitochondrial oxidative phosphorylation rate in participants with severe vitamin D deficiency. • Correction of vitamin D deficiency in these individuals led to noticeable improvement in symptoms of muscle weakness and fatigue.

c. Nutritional and pharmacological interventions with potential to improve mitochondrial health in women and prevent disease progression:

Nutrition plays an important role in promoting health by influencing mitochondrial function, a process increasingly recognized as central to the development and progression of various diseases. Several nutritional and pharmacological interventions have been reported to modulate mitochondrial function. However, the extent to which these mechanistic effects translate into clinically meaningful outcomes remains to be fully established. This is particularly relevant for women, who may experience alterations in mitochondrial function during physiological transitions such as reproduction, pregnancy, and menopause. For instance, vitamin D has been shown to enhance mitochondrial oxidative phosphorylation, increase VDR expression, and reduce inflammatory cytokines, mechanisms central to the pathophysiology of many diseases, which this vitamin can favorably modulate [56, 61]. Similarly, nicotinamide mononucleotide (NMN) restores impaired mitochondrial fission, enhances mitochondrial membrane potential, and improves mitochondrial respiration, while also improving insulin sensitivity and signaling in skeletal muscle [72–74]. In addition to the previously discussed interventions, Japanese functional foods, cabergoline, and MitoQ have also demonstrated protective effects on mitochondrial health [59, 66]. A detailed summary of all these studies is provided in Table 3. Overall, these studies demonstrate that nutritional and pharmacological interventions, ranging from vitamins and antioxidants to NAD^+^ precursors, functional foods, and compounds such as MitoQ and cabergoline, can support mitochondrial health. By promoting energy production, reducing oxidative stress, and enhancing key cellular pathways, these strategies hold real potential to help prevent or slow disease, particularly for women who are more susceptible to mitochondrial dysfunction at various stages of life.

**Table 3:** Nutritional and pharmacological interventions with potential to improve mitochondrial health in women and prevent disease progression.

Sr. No	Study ID	Intervention type	Intervention	Dosage	Impact on mitochondrial function
1.	Edzeamey et al. [65]	Nutrition/Pharmacological agent	• Dimethyl fumarate (DMF) • N‐acetyl cysteine (NAC) • L‐Ascorbic acid (LAA)	• Dimethyl fumarate (DMF)‐ 30 μM • N‐acetyl cysteine (NAC)‐ 100 μM L‐Ascorbic acid (LAA)‐ 20 μM	• Increases the mRNA expression level of FXN & NRF2 in human FRDA fibroblast. • Antioxidant interventions, particularly the combination of L‐ascorbic acid and N‐acetyl cysteine, effectively mitigate key cellular and mitochondrial abnormalities associated with FRDA. • These treatments not only reduced oxidative stress but also enhanced mitochondrial biogenesis, restored antioxidant defense pathways through NRF2 activation, and improved frataxin expression.
2.	Zhang et al. [72]	Nutrition	β‐Nicotinamide mononucleotide (NMN) (NAD + precursor)	NMN: 0.5 mM (in vitro)	NMN supplementation restored NAD + levels, suppressed mitochondrial fission, improved mitochondrial membrane potential (MMP) and respiration, and promoted apoptosis in OLP T cells.
3.	Vine et al. [66]	Nutrition/Vitamin	Thiamin and coenzyme Q10 (CoQ10)	• Thiamin:0.5 ug ml • CoQ10:1 ug ml	Thiamin and CoQ10 improve mitochondrial respiration, especially in patients with DKA, suggesting their potential to help restore mitochondrial energy metabolism in this condition.
4.	Phillips et al. [56]	Vitamin	Calcitriol	• Calcitriol of concentration: 10 and 100 nM (24 h)	Vitamin D supplementation in obese mothers improves mitochondrial respiration, enhances VDR expression, and reduces the production of inflammatory cytokines, such as IL‐18, in placental cells.
5.	Ekici et al. [59]	Pharmacological agent	Cabergoline (CBG)	0.5 mg of CBG tablet for 6 months	Treatment of neutrophils with the antioxidant CBG decreased ENDO‐induced TRPM2 channel activity, likely by reducing excessive ROS production.
6.	Sinha et al. [61]	Vitamin	Cholecalciferol	20,000 IU on alternate days for 10–12 weeks	Enhancement in the mitochondrial oxidative phosphorylation rate after the cholecalciferol therapy.
7.	Murray et al. [75]	Nutrition	MitoQ (mitochondrial‐targeted antioxidant)	Given for the period of 6 weeks	The excessive production of reactive oxygen species plays a key role in the pathophysiology of age‐related vascular endothelial dysfunction. Findings indicate that chronic supplementation with MitoQ leads to significant improvements in vascular endothelial function in older adults, primarily through reductions in circulating oxidized low‐density lipoprotein and enhancement of nitric oxide bioavailability. These changes are accompanied by decreased mitochondrial oxidative stress in endothelial cells, indicating that MitoQ exerts its beneficial effects by modifying the plasma environment and directly supporting cellular antioxidant defenses.
8.	Marotta et al. [73]	Nutrition	Japanese Functional Food (FPP)	4.5 g 2 times a day	Postmenopausal women experience increased oxidative stress and mitochondrial dysfunction, as shown by raised levels of malondialdehyde and carbonyl proteins, and reduced redox enzymes and BDNF (Brain‐derived neurotrophic factor). Supplementation with FPP led to improvements in antioxidant status and restoration of mitochondrial function in peripheral blood cells, even in women with elevated BMI. These findings highlight FPP as a promising intervention in managing menopause‐related mitochondrial and neurodegenerative risk, suggesting that broad antioxidant therapy is much less effective than targeted nutraceutical approaches.
9.	Yoshino et al. [74]	Nutrition	Nicotinamide mononucleotide (NMN)	• Period: 10 weeks of treatment • Dosage: 250 mg/day	NMN supplementation improved insulin sensitivity and signaling in skeletal muscle. NMN enhanced insulin‐stimulated phosphorylation of key proteins such as AKT and mTOR in muscle tissue, along with upregulation of genes involved in muscle remodeling, particularly those related to the platelet‐derived growth factor receptor β (PDGFRβ) pathway. Although NMN did not change overall muscle NAD + levels or affect body composition, liver, or adipose tissue insulin sensitivity, it increased the turnover of NAD + metabolites in muscle.

## 4. Quality Assessment/Risk of Bias Assessment

Given the varied methodological designs of the studies included in this review, we used quality appraisal tools tailored to each study type. For observational, clinical, and mixed‐method studies, we employed the Mixed Methods Appraisal Tool (MMAT) [76], which facilitates a systematic evaluation of qualitative, quantitative, and mixed‐method research designs. The tool assesses the methodological integrity across various areas, including the sampling strategy, measurement validity, thoroughness of outcome data, and potential biases. Among the 35 studies evaluated, a substantial majority satisfied most of the methodological criteria set forth by the MMAT. Almost all studies effectively measured both exposures and outcomes and applied appropriate statistical analyses to address their respective research questions. Specifically, in quantitative descriptive studies, a significant proportion employed relevant sampling strategies and reported samples that were representative of the target population. However, a small number of studies reported potential limitations related to sampling representativeness, nonresponse bias, or incomplete adjustment for confounding variables in the design or analysis. In some quantitative non‐randomized studies, the control for confounding factors was not explicitly detailed. Overall, the methodological appraisal suggested that the included studies were of moderate to high methodological quality, supporting the reliability of the evidence synthesized in this review.

Experimental research, encompassing in vitro studies, animal models, and laboratory mechanistic investigations, was assessed using the OHAT Risk of Bias Tool [77]. This framework assesses potential sources of bias across several domains, including selection bias, performance bias, detection bias, attrition bias, and selective reporting. Each included study was independently assessed according to the criteria specified within the respective appraisal tools. Based on these assessments, studies were categorized by methodological quality and risk of bias. The results of the quality appraisal were incorporated into the data synthesis to ensure that studies of acceptable methodological rigor supported the conclusions drawn from the evidence. Most studies maintained consistent experimental conditions across groups, thereby reducing the risk of performance bias. Attrition bias and selective reporting bias were also generally rated as low, indicating that most studies accounted for all samples and reported outcomes adequately. A few studies showed unclear detection or performance bias, mainly due to limited reporting of blinding procedures or experimental conditions. Overall, the included experimental studies demonstrated a low risk of bias, supporting the reliability of the mechanistic evidence on mitochondrial function and its interaction with nutritional factors and vitamins. A summary of the quality appraisal for all included studies is presented in the Supporting Information. The studies analyzed exhibit a minimal risk of bias.

## 5. Discussion

Ovarian aging and the transitions from perimenopause and postmenopause predispose women to various disorders. Ovarian aging not only results in diminished fertility and the onset of menopause but also predisposes women to systemic health issues, including cardiovascular disease, osteoporosis, cognitive decline, and metabolic disorders. Mitochondrial dysfunction is involved in most of the pathophysiology of the condition, impairing cellular energy production, increasing oxidative stress, and driving chronic inflammation.

### 5.1. Role of Mitochondria in the Pathophysiology of Metabolic and Endocrine Disorders

#### 5.1.1. Obesity

A heterogeneous condition arising from the complex interplay of age, sex, socioeconomic status, environmental, and biological factors [78]. This condition exhibits a higher prevalence in females (40%) than in males (35%) [79]. Obesity affects women across all ages, but its health consequences differ markedly depending on menopausal status due to mitochondrial dysfunction and hormonal changes. Likewise, during the reproductive years, women enter an important phase where fertility, maternal health, and child outcomes are of utmost importance [80]. Obesity can negatively influence all of these aspects, and current data indicate that over 40% of women with obesity fall within the 20–39‐year age group [81]. While hormonal activity during the reproductive years provides a protective influence, obesity may negate these effects. The menopausal transition is characterized by an increased risk of obesity, a shift from a gynoid to an android body shape, and a notable increase in abdominal and visceral fat deposition [82]. Menopause is accompanied by significant changes in physiological and metabolic pathways due to a deficiency of the key hormone estrogen. This affects lipid metabolism, reduces energy expenditure, and insulin resistance [83, 84]. Various factors can contribute to obesity, including the menopausal transition, environmental influences, and other causes. An imbalance between calorie intake and expenditure causes obesity [85], triggering adaptive responses in adipose tissue, primarily through adipocyte hypertrophy and hyperplasia [86]. This leads to enhanced storage of fatty acids within adipocytes [87]. In addition to their primary role of storing energy in the form of fat, adipocytes also produce bioactive molecules known as adipokines, which have important immunoregulatory properties. These adipokines participate in modulating inflammation, immune responses, and metabolism, thereby influencing systemic physiological and pathophysiological processes beyond simple energy storage [88, 89]. Inflammation and obesity are closely linked [90], as increased inflammatory responses significantly contribute to the production of ROS [91]. The body manages this oxidative stress through its antioxidant defense system, which comprises both enzymatic antioxidants, such as superoxide dismutase and catalase, and non‐enzymatic antioxidants like vitamins C and E [92]. This balance between ROS generation and antioxidant activity is crucial for maintaining cellular health and preventing obesity‐related complications.

#### 5.1.2. PCOS

A complex condition resulting from reproductive, endocrine, and metabolic abnormalities [93]. The prevalence of this disorder is estimated to range from ~4% to 18% worldwide among women of reproductive age [94]. Mitochondria play a crucial role in the pathophysiology of PCOS. PCOS primarily affects the ovaries, which consist of both somatic cells and germ cells. This dual involvement means that PCOS impacts not only ovarian function in the parent, but also can impact the health of the offspring [95]. Specifically, PCOS results in a significant reduction in ATP production, a decrease in mitochondrial membrane potential, lower citrate synthase activity, and a decreased oxygen consumption rate. These changes reflect impaired bioenergetics and a compromised ability of mitochondria to sustain critical cellular processes within the ovary, ultimately contributing to poor oocyte quality and reproductive dysfunction [96, 97]. Apart from impairing mitochondrial energetics, increased exposure of the developing ovary to PCOS is associated with elevated mitochondrial oxygen consumption and enhanced oxidative stress that persists across the lifespan. Similar to its effects on the ovary, PCOS exposure is known to disrupt mitochondrial function in granulosa cells, the placenta, the uterus, and oocytes [95]. Therapeutic approaches for PCOS range from lifestyle modifications, such as balanced nutrition and increased physical activity, to targeted pharmacological and nutritional interventions aimed at restoring mitochondrial function. Nutritional interventions represent an accessible and cost‐effective strategy that can be easily integrated into daily routines to promote healthier aging, particularly in women with PCOS who face increased risks of metabolic and reproductive complications. Several nutrients and supplements, including vitamin D, vitamin C, vitamin E, MitoQ, and NAD^+^ precursors, have shown promising effects in reducing oxidative stress, enhancing mitochondrial function, and restoring cellular homeostasis [98]. By targeting mitochondrial pathways, these interventions not only support energy metabolism but may also help mitigate systemic inflammation and improve long‐term health outcomes.

### 5.2. Role of Mitochondria in the Pathophysiology of Disorders Involving the Central Nervous System

As women approach reproductive senescence and transition through menopause, they not only experience the cessation of ovarian function but also undergo neuroendocrine aging, characterized by alterations in the hypothalamic pituitary gonadal axis, changes in hormonal regulation, and increased vulnerability to age‐related metabolic and neurological disorders [99]. The decline in estrogen during the menopausal transition has profound effects on neuronal processes, acting through genomic or nongenomic pathways [99]. The transition from puberty to the perimenopausal state spans a long time interval and involves complex, interrelated physiological pathways [100]. Because these transitional phases are multifactorial, disturbances to endogenous or exogenous factors can trigger new vulnerabilities or reveal already existing ones during the restructuring process [101]. The transition from a healthy state to pre‐disease and then to established disease is a complex continuum, and in neurological disorders, early signs during the perimenopausal stage can serve as significant warnings. During the perimenopausal stage, women may begin to experience subtle but important changes, including insomnia, mood disturbances, memory lapses, and cognitive difficulties. These early signs can serve as warning signals, highlighting an increased susceptibility to neurodegenerative diseases such as Alzheimer’s, a risk that becomes more pronounced after menopause [102, 103].

#### 5.2.1. AD

AD is more prevalent in women than in men, with about two‐thirds of all clinically diagnosed cases occurring in females, highlighting a clear sex‐based disparity in susceptibility [104]. Evidence indicates that mitochondrial dysfunction plays a significant role in the pathophysiology of this condition [105]. Importantly, mitochondrial dysfunction may manifest before any overt neuropathological symptoms, suggesting it could act as an early warning sign in the disease process [106]. Additionally, mtDNA‐encoded COX subunits are selectively compromised in the frontal cortex of females with AD. Importantly, this impairment is observed only in mtDNA‐encoded COX subunits and not in those encoded by nuclear DNA [107]. Menopause is marked by a decline in estrogen levels, a hormone essential for maintaining brain energy metabolism, synaptic plasticity, and cognitive function. The reduction in estrogen disrupts these critical processes, leading to decreased metabolic activity, accumulation of amyloid‐beta plaques, and increased inflammation in the brain. These changes impair neuronal health and connectivity, contributing to cognitive decline [108]. Approximately 70% of AD cases occur in postmenopausal women [109]. AD ranks as the fifth leading cause of death among individuals aged 65 and older, underscoring its significant impact on the elderly population [110]. This growing public health concern necessitates increased attention toward effective therapeutic strategies. Current interventions include hormonal therapies aimed at restoring estrogen function, which plays a key role in maintaining brain health. Alongside hormone therapy, nutritional supplementation is also employed to support cognitive function and overall brain health, offering potential improvements over time [111]. Adhering to the Mediterranean Diet (MD), Dietary Approaches to Stop Hypertension (DASH), and Mediterranean‐DASH Intervention for Neurodegenerative Delay (MIND) diets has been shown to reduce the future risk of AD [112]. Diets rich in antioxidants may contribute to improved neuronal and mitochondrial health; however, the extent to which these dietary patterns influence disease progression remains to be fully established. Additionally, increasing intake of vitamins A, B, C, D, and E in the regular diet can help prevent the development of AD [113].

### 5.3. Role of Mitochondria in the Pathophysiology of Cardiovascular Disorders (CVD)

CVD encompass a broad spectrum of conditions affecting the heart and blood vessels, including coronary artery disease (CAD), cerebrovascular disease, peripheral arterial disease, rheumatic heart disease, aortic atherosclerosis, and others [114]. CVD is one of the leading causes of mortality in women, accounting for one death every 8 s globally [115]. The risk of CVD significantly increases after the fifth decade of life, coinciding with the menopausal stage in women [116]. Healthy cardiomyocytes rely heavily on mitochondrial oxidative phosphorylation to generate the energy required for contractile function. During development, cardiomyocytes primarily use glucose, but in adulthood, they shift to relying on fatty acid oxidation to meet their high energy requirements [117]. Sex hormones have a predominant role in maintaining overall mitochondrial function within cardiomyocytes [117]. With the decline in estrogen during menopause, women become more vulnerable to cardiovascular disease. Mitochondria, which are critical for generating the energy required by cardiomyocytes, are also the primary producers of ROS. Excess ROS can drive inflammation, endothelial dysfunction, and other detrimental effects, amplifying cardiovascular risk [118]. Failure of the reactive oxygen species scavenging system can exacerbate complications in cardiovascular disease. Given the crucial role of oxidative stress in CVD, therapeutic strategies targeting both ROS production and enhancing ROS scavenging systems are emerging as promising approaches, including compounds such as MitoTEMPO, mitoQ, and SS‐31/MPT‐131 [119].

### 5.4. Role of Mitochondria in the Pathophysiology of Muscle‐Related Disorders

Musculoskeletal health becomes a major concern for women during midlife and beyond, particularly after menopause. These changes significantly increase the risk of locomotor disability, frailty, falls, and fractures, which collectively worsen quality of life and elevate mortality risk in postmenopausal women [120]. Menopause predisposes women to several musculoskeletal conditions, including osteoporosis, sarcopenia, and osteoarthritis (OA).

#### 5.4.1. Osteoporosis

Osteoporosis is a condition characterized by decreased bone mineral density and bone mass, resulting in bones that are weaker and more susceptible to fractures. This reduction in bone density alters bone structure and strength, making bones more susceptible to fractures [121]. Under normal physiological conditions, mitochondria play a central role in maintaining calcium flux and homeostasis, and in regulating apoptosis by virtue of their characteristic functions of fusion and fission [122]. Dynamic‐related protein 1 (DRP1) is a protein that is involved in the mitochondrial fission function [123]. The risk of osteoporosis is usually elevated when mitochondrial fusion and fission are altered, potentially disrupting the activities of osteoblasts and osteoclasts, the cells responsible for bone formation and resorption. Increased oxidative stress has been reported to induce osteoblast dysfunction via DRP1‐mediated mitochondrial fission, potentially impairing mitochondrial morphology and function. Such disturbances in mitochondrial dynamics may contribute to reduced bone formation and increased bone resorption, thereby potentially contributing to bone loss and the development of osteoporosis [124].

#### 5.4.2. OA

OA, a common joint disorder, involves progressive degeneration of cartilage, secondary bone hypertrophy, and results in pain and functional disability [125]. The condition is especially prevalent in individuals around the age of 50 [126]. Articular cartilage chondrocytes, which are highly glycolytic [127], also rely on mitochondrial function to meet their energy demands and maintain cellular homeostasis. In OA, mitochondrial dysfunction is a key factor in disease pathogenesis, arising in part from somatic mutations in mtDNA within chondrocytes. This dysfunction impacts multiple cellular pathways, including increased oxidative stress, impaired biosynthetic processes, enhanced chondrocyte apoptosis, and cytokine‐induced inflammation that promotes matrix catabolism and cartilage matrix calcification. Mitochondrial respiratory chain (MRC) activity and ATP synthesis are also compromised, further impairing chondrocyte function. The resulting imbalance contributes significantly to cartilage degradation, inflammation, and the progression of OA [128].

#### 5.4.3. Sarcopenia

Sarcopenia is a musculoskeletal disorder characterized by a gradual decline in muscle mass and strength with aging [129]. During the menopausal transition, women experience an accelerated loss of lean body mass at ~0.5% per year. Compared to postmenopausal women, premenopausal women tend to have higher lean muscle mass [130]. The prevalence of sarcopenia in postmenopausal women varies by age group: it is ~1.4% in those aged 60–69 years, increases to 4.9% among women aged 70–79 years, and rises further to 12.5% in women aged 80 years and older. This age‐related increase highlights the progressive nature of muscle loss with advancing age in postmenopausal women [131]. The development of sarcopenia involves multiple pathogenic mechanisms, including mitochondrial dysfunction and oxidative stress [132, 133]. With aging, mitochondrial quality control mechanisms decline, leading to the accumulation of damaged mitochondria, increased ROS production, and oxidative stress. These mitochondrial defects impair muscle cell function, contributing to muscle fiber atrophy and degeneration. Furthermore, the accumulation of mtDNA mutations over time compromises the efficiency of the electron transport chain, reducing ATP synthesis and further disrupting muscle energy metabolism [134]. Therefore, maintaining mitochondrial health may be important for preserving muscle integrity during aging, although further studies are required to clarify its role in the development and progression of sarcopenia.

## 6. Conclusion

The evidence reviewed here highlights the potential of nutrition to modulate mitochondrial health, which may help mitigate some of the adverse effects of ovarian aging. Nutritional interventions that enhance mitochondrial function may confer beneficial biological effects and improve health in aging women; however, further well‐designed human studies are required to determine their impact on disease risk. Future research should focus on dietary modulation of mitochondrial pathways to develop effective, evidence‐based strategies to support women’s health during and beyond the menopausal transition.

## Author Contributions

**Kunjal Kiran Pai**: conceptualization, protocol development, search strategy design, literature identification, screening, and data extraction. **Shambhavi Shetye**: conceptualization, protocol development, search strategy design, screening, data extraction, manuscript drafting, and revision. **Rakshith Patil**: critical review and approval of the final manuscript. **Shravya Acharya**: conceptualization, protocol development, search strategy design, screening. **Kishan S. Kulal**: conceptualization, protocol development, search strategy design, screening, and critical review. **Ajeetkumar Patil**: critical review and approval of the final manuscript. **Saritha U. Kamath**: conceptualization, final review, and overall supervision of the manuscript.

## Funding

The authors have nothing to report.

## Disclosure

After using ChatGPT and Grammarly, the authors reviewed and edited the content as needed and take full responsibility for the content of the published article.

## Ethics Statement

The authors have nothing to report.

## Consent

The authors have nothing to report.

## Conflicts of Interest

The authors declare no conflicts of interest.

## Supporting Information

Additional supporting information can be found online in the Supporting Information section.

## Supporting information


**Supporting Information** The supporting files contain a table of the quality appraisal for all included studies.

## Data Availability

The authors have nothing to report.
