# Supplementary material for: Interplay of Mitochondrial Dynamics, Nutrition, and Vitamins: Evidence From Experimental and Human Studies With Implications for Women’s Health
Source: Oxid Med Cell Longev. 2026 Jun 26;2026:8685788. doi: 10.1155/omcl/8685788 (PMC13305678; doi:10.1155/omcl/8685788)
Supplement: Supplementary file 1 — Supporting Information The supporting files contain a table of the quality appraisal for all included studies. [file OMCL-2026-8685788-s001.docx]

Critical appraisal of observational and descriptive human research

| **Study ID / Author-Year** | Are there clear research questions? | Do the collected data allow to address the research questions? | **Quantitative non- randomized** | | | | | **Quantitative descriptive** | | | | |
| --- | --- | --- | --- | --- | --- | --- | --- | --- | --- | --- | --- | --- |
|  |  |  | Are the participants representative of the target population? | Are measurements appropriate regarding both the outcome and intervention (or exposure)? | Are there complete outcome data? | Are the confounders accounted for in the design and analysis? | During the study period, is the intervention administered (or exposure occurred) as intended? | Is the sampling strategy relevant to address the research question? | Is the sample representative of the target population? | Are the measurements appropriate? | Is the risk of nonresponse bias low? | Is the statistical analysis appropriate to answer the research question? |
| Lasse et al. (2021) | Yes | Yes |  |  |  |  |  | Yes | Yes | Yes | Yes | Yes |
| Farinha et al. (2014) | Yes | Yes |  |  |  |  |  | Yes | Yes | Yes | Yes | Yes |
| Martucci et al. 2020 | Yes | Yes |  |  |  |  |  | Yes | Yes | Yes | Yes | Yes |
| Yeh et al. (2005) | Yes | Yes |  |  |  |  |  | Yes | Yes | Yes | Yes | No |
| Warren et al. (2017) | Yes | Yes |  |  |  |  |  | Yes | Yes | Yes | Yes | Yes |
| Shaik et al. (2011) | Yes | Yes |  |  |  |  |  | Yes | Yes | Yes | Yes | Yes |
| Liu et al. (2014) | Yes | Yes |  |  |  |  |  | Yes | Yes | Yes | Yes | Yes |
| Rubio et al. (2018) | Yes | Yes |  |  |  |  |  | Yes | Yes | Yes | Yes | Yes |
| Ward et al. (2022) | Yes | Yes |  |  |  |  |  | Yes | Yes | Yes | Not sure | Yes |
| Jakus et al. (2017) | Yes | Yes |  |  |  |  |  | Yes | Yes | Yes | Yes | Yes |
| Ronkainen et al.(2010) | Yes | Yes |  |  |  |  |  | Yes | Yes | Yes | No | Yes |
| Lempesis et al. (2024) | Yes | Yes |  |  |  |  |  | Yes | Yes | Yes | No | Yes |
| Mosconi et al. (2021) | Yes | Yes |  |  |  |  |  | Yes | Yes | Yes | Yes | Yes |
| Manfredi et al. (2013) | Yes | Yes |  |  |  |  |  | Yes | Yes | Yes | Yes | Yes |
| Dravish et al. (2025) | Yes | Yes |  |  |  |  |  | Yes | Yes | Yes | Yes | Yes |
| Hima et al. (2015) | Yes | Yes |  |  |  |  |  | Yes | Yes | Yes | Yes | Yes |
| Vinothini et al. (2011) | Yes | Yes |  |  |  |  |  | Yes | Yes | Yes | Yes | Yes |
| Razmara et al. (2008) | Yes | Yes |  |  |  |  |  | Yes | Yes | Yes | Yes | Yes |
| Carvalho et al. (2024) | Yes | Yes |  |  |  |  |  | Yes | Yes | Yes | Yes | Yes |
| Sastre-Serra et al. (2013) | Yes | Yes |  |  |  |  |  | Yes | Yes | Yes | Yes | Yes |
| Adams-Reyes et al. (2019) | Yes | Yes |  |  |  |  |  | Yes | Yes | Yes | Yes | Yes |
| Qin et al. (2024) | Yes | Yes |  |  |  |  |  | Yes | Yes | Yes | Not sure | Yes |
| Phillips et al. (2022) | Yes | Yes |  |  |  |  |  | Yes | Yes | Yes | Yes | Yes |
| Kaushal et al. (2022) | Yes | Yes |  |  |  |  |  | Yes | Yes | Yes | Yes | Yes |
| Palacka et al. (2021) | Yes | Yes | Yes | Yes | Yes | No | Yes |  |  |  |  |  |
| Ekici et al. (2020) | Yes | Yes | Yes | Yes | Yes | No | Yes |  |  |  |  |  |
| Gong et al. (2020) | Yes | Yes | Yes | Yes | Yes | No | Yes |  |  |  |  |  |
| Vernerova L et al. (2025) | Yes | Yes | Yes | Yes | Yes | Yes | Yes |  |  |  |  |  |
| M. Nemec et al. (2021) | Yes | Yes | Yes | Yes | Yes | No | Yes |  |  |  |  |  |
| Sunitha B et al. (2016) | Yes | Yes | Yes | Yes | Yes | No | Yes |  |  |  |  |  |
| Taneera et al. (2025) | Yes | Yes | Yes | Yes | Yes | No | Yes |  |  |  |  |  |

Critical appraisal of experimental and mechanistic studies

| **Study ID / Author-Year** | **Disease / Model** | **Selection Bias (group comparability/randomization)** | **Performance Bias (identical conditions except intervention)** | **Detection Bias (blinding/objective measurement)** | **Attrition Bias (all samples accounted for)** | **Selective Reporting Bias** | **Other Bias / Confounding (cell line, passage, culture conditions)** | **Overall Risk of Bias** |
| --- | --- | --- | --- | --- | --- | --- | --- | --- |
| Edzeamey et al. (2025) | Friedreich’s Ataxia (FRDA)/Healthy Human Fibroblast GM23976 | Low | Low | Low | Low | Low | Low | Low |
| Zhang et al. (2025) | Oral lichen planus (OLP)/T cells | Probably Low | Low | Low | Low | Low | Low | Low |
| Vine et al. (2024) | Diabetic ketoacidosis/peripheral blood mononuclear cells (PBMC)/Blood samples | Low | Low | Low | Low | Low | Low | Low |
| Phillips et al. (2022) | Obesity/maternal and cord blood, placental blood | Low | Low | High/unclear | Low | Low | Low | Low |
| Ekici et al. (2020) | Endometriosis/Human neutrophils | Low | Low | High/unclear | Low | Low | Low | Low |
| Sinha et al. (2013) | Vitamin D Deficiency/Blood | Low | Unclear | Low | Low | Low | Low | Low |
| Murray et al. (2023) | Endothelial cell function and mitochondrial oxidative stress/Plasma | Low | Unclear | Low | Low | Low | Low | Low |
| Marotta et al. (2020) | Neurodegenartive disease/Blood | Low | Low | Low | Low | Low | Low | Low |
| Yoshino et al. (2021) | Obesity and prediabetes/Blood | Low | Low | Low | Low | Low | Low | Low |
